# Comparisons in polypharmacy over a decade in community-dwelling older adults-findings from Israel national health and nutrition surveys

**DOI:** 10.1186/s12877-022-03171-8

**Published:** 2022-06-13

**Authors:** Rebecca Goldsmith, Rita Dichtiar, Tal Shimony, Lesley Nitsan, Rachel Axelrod, Irit Laxer-Asael, Iris Rasooly, Tali Sinai, Elliot M. Berry

**Affiliations:** 1 Braun School of Public Health, The Hebrew University of Jerusalem, 9112001 Jerusalem, Israel; 2 Israel Center for Disease Control, Israel Ministry of Health, 5262100 Ramat Gan, Israel; 3 Geriatric Division, Israel Ministry of Health, 39 Yirmiyahu St, 9101002 Jerusalem, Israel; 4 School of Nutritional Sciences, The Robert H. Smith, Faculty of Agriculture, Food and Environment, The Hebrew University of Jerusalem, 7610001 Rehovot, Israel

**Keywords:** Polypharmacy, Elderly, Medications, Aging, Mabat, Survey

## Abstract

**Background:**

Polypharmacy increases with age and is associated with serious health and economic costs. This study reports changes over a decade in medication-use patterns and polypharmacy, in Israeli community-dwelling older adults aged ≥ 65 years.

**Methods:**

Demographic and health data from two representative national health cross-sectional surveys – MABAT ZAHAV 1 (MZ1) in 2005–2006, and MZ2 in 2014–2015 were analyzed. Polypharmacy was defined as use of ≥ 5 medications. Risk factors for polypharmacy were estimated by multivariable logistic regression with adjusted odds ratios (aOR) and their 95% confidence intervals (CI).

**Results:**

Self-reported data on medications taken were available for 1647 participants (91.5%) in MZ1, and for 833 participants (80.2%) in MZ2, 55% women, and about 20% aged ≥ 80, in both surveys. The prevalence of polypharmacy was significantly lower in MZ2 than in MZ1: 64.2% versus 56.3%, p = .0001; with an aOR (95%CI) of 0.64 (0.52, 0.80). The most commonly taken drugs were for hypertension (27.0%, 25.3%), dyslipidemia (9.7%, 12.4%) and anticoagulation (9.2%, 9.8%). For approximately 10% of drugs, indications were either unknown or incorrect. Polypharmacy was significantly associated with poor self-health assessment 2.47 (1.99, 3.06), ≥ 4 versus 1–3 chronic illnesses 6.36 (3.85, 10.50), and age ≥ 80 versus younger 1.72 (1.32, 2.24). Similar associations were observed with major polypharmacy of ≥ 8 medications.

**Conclusion:**

Polypharmacy, although reduced in the last decade, requires constant attention, especially concerning lack of knowledge of indications which leads to poor adherence and adverse side effects. Health-care teams should carry out regular medicine reconciliation in at-risk elderly patients.

## Background

One of the major achievements of modern medicine and public health is prolonging life expectancy, thereby increasing the size of the aging population. Elderly people have more chronic conditions and disabilities, use more healthcare services and hence require an increasing number of drugs, referred to as "polypharmacy" [1–3]. Polypharmacy increases the probability of inappropriate prescribing, taking the wrong medication, cascade prescribing of side effects, and poor adherence-all of which increase the risk for adverse drug events [4], hospitalizations, poor functional status, morbidity and mortality [5, 6].

Polypharmacy as a screening measure is relatively easy to obtain, and provides an indication of the extent of medication burden. There is a need to address the issue in the clinical setting. Several threshold levels e.g., more than 5, 6 or 8 medications, and a variety of criteria, e.g. Beers criteria, STOPP/START criteria, have been used [7]. A systematic review of the definitions of polypharmacy showed that routinely taking five or more medications daily was most commonly applied [8]. Furthermore, there are inconsistencies whether to include non-prescribed medication (over-the-counter, OTC) and dietary supplements. Thus, comparing countries and conducting longitudinal studies within a specific country are very challenging.

Globally, polypharmacy, especially in older adults, is a widespread and quite complex phenomenon, with prevalence between 27%-59% in primary care patients, and 46%-84% in hospital care [9]. According to data from the National Health and Nutrition Examination Survey (2015–2016) and the Canadian Health Measures Survey (2016–2017), 34.5% and 30.9% of adults aged 60–79 in the United States and in Canada, respectively, used at least 5 prescription drugs [10]. The prevalence of polypharmacy (use of five or more medications) ranged from 26.3% to 39.9% in community-dwelling adults aged 65 or more years who participated in Wave 6 of the Survey of Health, Ageing, and Retirement in Europe (SHARE), including 17 countries [11].

In 2020, Israel’s population reached 9.2 million people with those aged 65 and over representing 12.0%, of those 40.2% above age 75 [12]. However, the data regarding polypharmacy in Israel are limited [13], and those supplied by the four Health Maintenance Organizations (HMOs), covering all of the population reports were not uniform regarding parameters such as medical and sociodemographic profiles, hence giving a problematic wide range of polypharmacy between 7.3% and 51% among those aged 75 + who took 8 or more medications [14]. Despite the documented prevalence of polypharmacy, there were no policy changes regarding medication prescribing and/or dispensing. In light of these publications, it is a necessary public health priority to examine both the scope of the phenomenon and its trends, based on uniform measurement tools, namely using identical definitions, means used to document medication usage (direct observation vs. reported usage). The objectives of this study were to describe and compare drug-taking patterns in Israel from two nationally representative health surveys a decade apart, to estimate the extent of polypharmacy in community-dwelling older adults, and associated factors.

## Methods

This study analyzed data from two Israeli National Health and Nutrition Surveys of the Elderly (MABAT Zahav)—representative, cross-sectional surveys of the Israeli community-dwelling population aged 65 and over. The first survey—Mabat Zahav 1 (MZ1) was carried out between the years 2005–2006, and the second—Mabat Zahav 2 – (MZ2) in 2014–2015, by the Israel Center for Disease Control together with the Department of Nutrition (both part of the Ministry of Health), and with the cooperation of other health organizations in Israel including the Israeli Central Bureau of Statistics (CBS) (MZ2 only) and two of the Health Maintenance Organizations (MZ1 only). Survey design and operation have been described in detail elsewhere [15–17]. Briefly, a national stratum sampling was performed, according to population group (Jews/Arabs) and locality. In MZ 1 and MZ2, 1852 and 1039 older adults, respectively, signed informed consent, were interviewed face- to- face, by survey personnel. Interviews focused on demographic, health status and lifestyle characteristics. The surveys questionnaires were pretested in pilot studies, and construct validity was also carried out. The questionnaires have been described in detail elsewhere [15, 16].

### Study Population

Of the 1852 and 1039 participants in MZ1 and MZ2 surveys, 46 and 10 were excluded, respectively, because of significant cognitive impairment (i.e., less than 17 in the Mini Mental State Examination-MMSE, or they were unable to remember or concentrate) [15, 16]. A further 7 were excluded from MZ1, due to missing data. The survey data sets included 1799 (MZ1) and 1029 (MZ2).

For examination of drug taking patterns, those who were taking at least one drug on a regular basis, and were able to present to the interviewer the drugs/drugs packages, were included. The dataset in this study contained 1647 respondents (91.5%) from MZ1 and 833 respondents (80.2%) from MZ2.

### Assessment of medication use

The use of prescription and over- the- counter (OTC) drugs (including dietary supplements) was asked. In order to ensure correct listing and naming of drugs, the interviewees were requested to present to the interviewers all the drugs and drug packages they took on a regular basis (chronic medications). Temporary drugs, such as antibiotics or short-term analgesics, were excluded from the analyses. For each drug, subjects were asked about dosage, mode of delivery (oral, eye drops, creams etc.) and indication. Then, all drugs, including dietary supplements and OTC drugs, were coded using the WHO Anatomical Therapeutic Chemical (ATC) classification system [18].

The data presented are from those respondents taking regularly at least one medication, and who showed the medication packages for accurate recording of drug name, dose and manufacturer. Only a small percentage (1.2% MZ1, 6.5% MZ2) were unable to show the interviewer their medications. Polypharmacy was defined as the concurrent use of five or more drugs (including OTCs) per person [19, 20], and to describe the finding as defined in the Israeli State Comptroller's report, the use of eight or more drugs was defined in this study as major polypharmacy [14].

### Demographic and health characteristics definitions

Age (years) divided to two subgroups (65–79, 80 years and over); Population group defined based on the definitions used by the CBS as Jews/Arabs. Socioeconomic status (SES) was also defined according to the National Insurance Institute (NII), as based on income, and family size and presented as above or below the poverty line. Education level presented as four categories according to numbers of years of education, and if 13 or more, with or without an academic degree: (1) 0–8 years, (2) 9–12 years, (3) 13 years or more, not academic, (4) 13 years or more academic**.**

Subjective assessment of health status was defined according to self-report. Participants were asked about their health in general, and responses were classified dichotomously: 'good' or 'very good' health were classified as good, other responses were classified as poor. Comorbidity (number of diseases per person), was based on self- reporting on existence of chronic illnesses diagnosed by a physician, including heart diseases, stroke, asthma, diabetes, hypercholesterolemia, hypertension, and cancer.

### Statistical analysis

Categorical variable data are presented as n (%) and chi-square analysis was used to test for statistical significance. Continuous variables are presented as mean (SD) or median and interquartile range (IQR, 25^th^, 75^th^), and analyzed by a t-test or Mann Whitney U- test. Logistic regressions were used to examine the odds ratios (OR), 95% confidence interval (95% CI) of the outcome variables of polypharmacy (≥ 5 medications) and of major polypharmacy (≥ 8 medications) for possible health-related and socio-demographic factors. Multivariable models were performed and the covariates were: the survey, sex, age group, education, population group, socioeconomic status, subjective health status, comorbidity. Tests were 2-tailed, and statistical significance was set at *P* < 0.05. Analyses were performed using SAS statistical software (version 9.4).

### Ethical approval

The first survey received approval from the Ethics Committee of the Sheba Medical Center, and the second from the Israeli Ministry of Health. All survey participants from both surveys provided written informed consent and all guidelines of the Ethics committees were followed.

## Results

### Demographic and health characteristics of participants

Table 1 shows the demographic and health characteristics of participants according to survey. The two population samples were similar in sex and age groups distribution, with about 45% males and about fifth aged 80 years and over. In MZ2 survey the interviewees were significantly more educated, with a 10.6% difference in prevalence of academic participants (*n* = 248, 30.6% and 328, 20%, respectively) and a 7.5% difference in prevalence of having 0–8 years of education (206, 25.4% and 540, 32.9%, respectively). Generally, MZ2 participants were of a higher SES, with 526/650 (80.9%) above the poverty line as compared to 947/1286 (73.6%) in the earlier survey. The prevalence of interviewees reporting that their health status was good was higher in MZ1 versus MZ2: 878/1647 (53.3%) vs. 383/828 (46.3%). However, no significant differences were observed regarding prevalence of comorbidity between surveys.

**Table 1:** Demographic and health characteristics of older adults who use drugs regularly, according to survey

Characteristics	Mabat Zahav 1 *N* = 1647 n (%)	Mabat Zahav 2 *N* = 833 n (%)	P*
Sex			0.85
Males	748 (45.4)	375 (45.0)	
Females	899 (54.6)	458 (55.0)	
Age group, years			0.62
65–79	1284 (78.0)	642 (77.1)	
≥ 80	363 (22.0)	191 (22.9)	
Education, years			<0.0001
0–8 years	540 (32.9)	206 (25.4)	
9–12 years	528 (32.1)	230 (28.4)	
≥ 13 not academic	247 (15.0)	126 (15.6)	
≥ 13 academic	328 (20.0)	248 (30.6)	
Population group			<0.001
Jews	1384 (84.0)	743 (89.2)	
Arabs	263 (16.0)	90 (10.8)	
Socioeconomic status			<0.001
Above poverty line	947 (73.6)	526 (80.9)	
Below poverty line	339 (26.4)	124 (19.1)	
Subjective Health status			<0.001
Good	878 (53.3)	383 (46.3)	
Poor	769 (46.7)	445 (53.7)	
Comorbidity			0.14
No chronic illness	156 (9.6)	97 (11.7)	
1–3 chronic illnesses	1248 (76.5)	635 (76.3)	
≥ 4 chronic illnesses	228 (14.0)	100 (12.0)	
Polypharmacy			< .0001
(< 5 drugs/d)	589 (35.8)	364 (43.7)	
(≥ 5 drugs/d)	1058 (64.2)	469 (56.3)	
Major polypharmacy			< .0001
(< 8 drugs/d)	1154 (70.1)	644 (77.3)	
(≥ 8 drugs/d)	493 (29.9)	189 (22.7)	

^*^p-value for the differences between surveys (Mabat Zahav 1 and 2)

### Polypharmacy and associated factors

**Fig. 1:**
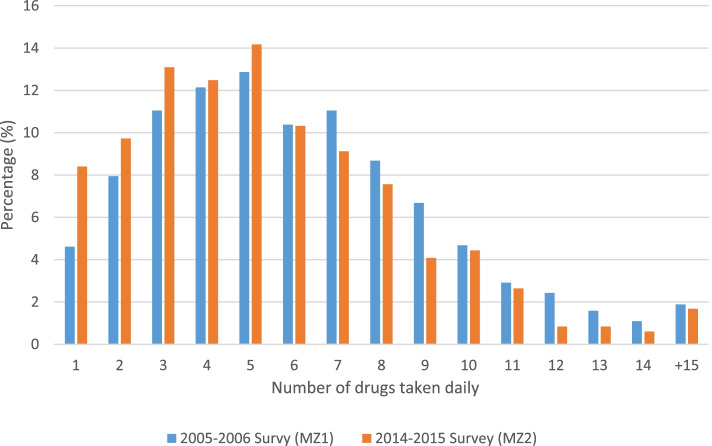
Prevalence of the number of drugs taken daily by person according to survey

**Fig. 2:**
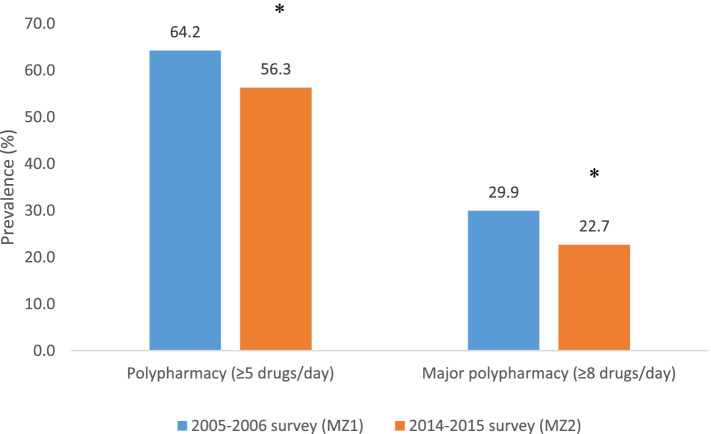
Prevalence of polypharmacy according to surveys**.** * *p* < .0001 between surveys

There was a higher number of drugs per day consumed regularly by individuals in MZ1 compared to MZ2 (median: 6, IQR: 4, 8 and 5, IQR: 3, 7, respectively, *p* < 0.0001). The frequency of drugs taken, according to survey is presented in Fig. 1. Taking 5 or more drugs regularly, which was defined as polypharmacy, was reported by 64.2% vs. 56.3% in MZ1 and MZ2, respectively, *P* = 0.0001; and major polypharmacy, defined as taking 8 medications or more, was reported by 29.9% and 22.7% of participants from MZ1 and MZ2, respectively, *p* = 0.0001 (Fig. 2). Based on the preliminary power calculations of the given study samples, 1647 and 833 in MZ1 and MZ2, respectively, the study power to detect the reported 7.9%, and 7.2% effect sizes (differences in polypharmacy and major polypharmacy prevalence between surveys) were very high (> 96.9%, and > 96.6% respectively).

The prevalence of polypharmacy according to demographic and health characteristics are presented in Table 2. In both surveys, a significantly higher prevalence of polypharmacy was found among the older age groups, those with poor subjective health status, with comorbidities. In MZ1, women had significantly higher prevalence of polypharmacy and in MZ2 also in those with less education. In both surveys, the association with low SES (below the poverty line) was not statistically significant, and no associations were found with population group. Similar results were observed for the crude associations of major polypharmacy and these factors, except for population group, where Jews had a higher prevalence of major polypharmacy in MZ2.

**Table 2:** The prevalence of polypharmacy and major polypharmacy in Israeli older adults according to demographic and health indicators in each survey

Characteristics	Polypharmacy (≥ 5 drugs) n (%)				Major-polypharmacy (≥ 8 drugs) n (%)			
	**MZ1**	***P* value**	**MZ2**	***P* value**	**MZ1**	***P* value**	**MZ2**	***P* value**
Sex		0.044		0.20		0.031		0.89
Males	461 (61.6)		202 (53.9)		204 (27.3)		86 (22.9)	
Females	597 (66.4)		267 (58.3)		289 (32.3)		103 (22.5)	
Age group, years		< .001		< .0001		0.004		< .0001
65–79	796 (62.0)		329 (51.3)		362 (28.2)		119 (18.5)	
≥ 80	262 (72.2)		140 (73.3)		131 (36.1)		70 (36.7)	
Education, years		0.61		< .001		0.38		< .0001
0–8 years	359 (66.5)		140 (68.0)		170 (31.5)		72 (35.0)	
9–12 years	333 (63.1)		120 (52.2)		163 (30.9)		50 (21.7)	
≥ 13 not academic	155 (62.8)		72 (57.1)		72 (29.2)		25 (19.8)	
≥ 13 academic	208 (63.4)		121 (48.8)		86 (26.2)		39 (15.7)	
Population group		0.21		0.061		0.031		0.67
Jews	898 (64.9)		410 (55.2)		429 (31.0)		167 (22.5)	
Arabs	160 (60.8)		59 (65.6)		64 (24.3)		22 (24.4)	
Socioeconomic status		0.20		0.07		0.46		0.06
Above poverty line	597 (63.0)		279 (53.0)		273 (28.8)		111 (21.1)	
Below poverty line	227 (67.0)		77 (62.1)		105 (31.0)		36 (29.0)	
Subjective Health status		< .0001		< .0001		< .0001		< .0001
Good	459 (52.3)		156 (40.7)		167 (19.0)		37 (9.7)	
Poor	599 (77.9)		311 (69.9)		326 (42.4)		152 (34.2)	
Comorbidity		< .0001		< .0001		< .0001		< .0001
No chronic illness	51 (32.7)		18 (18.6)		13 (8.3)		4 (4.1)	
1–3 chronic illnesses	785 (62.9)		362 (57.0)		340 (27.2)		131 (20.6)	
≥ 4 chronic illnesses	217 (95.2)		89 (89.0)		137 (60.1)		54 (54.0)	

*P* values—for differences in the prevalence of polypharmacy and major polypharmacy according to demographic and health characteristics in each survey

*MZ1* Mabat Zahav 1, *MZ2* Mabat Zahav 2 (Israeli National Health and Nutrition Surveys 2004–2005 and 2014–2015, respectively)

Multivariable logistic regression was used to evaluate the adjusted association between polypharmacy, survey and the health-related and sociodemographic covariates (Table 3). The prevalence of polypharmacy and major polypharmacy in 2014–2015 was significantly lower than in 2005–2006. Age 80 years and older, subjective health status, and a number of comorbidities were significantly associated with polypharmacy. Being female was associated with polypharmacy but not major polypharmacy.

**Table 3:** The association of factors with polypharmacy and major polypharmacy in Israeli older adults according to survey [Multivariable Models]

Characteristics	Polypharmacy (≥ 5 drugs)		Major polypharmacy (≥ 8 drugs)	
	OR (95%CI)	p	OR (95%CI)	p
Survey				
Mabat Zahav 1	1		1	
Mabat Zahav 2	0.64 (0.52—0.80)	< .0001	0.71 (0.56—0.90)	0.005
Sex				
Males	1		1	
Females	1.19 (0.97—1.50)	0.10	0.99 (0.80—1.24)	0.98
Age group, years				
65–79	1		1	
≥ 80	1.72 (1.32—2.24)	< .0001	1.44 (1.12—1.86)	0.005
Education, years				
0–8 years	1		1	
9–12 years	0.86 (0.65—1.14)	0.28	0.90 (0.68—1.21)	0.48
≥ 13 not academic	1.02 (0.72—1.44)	0.91	0.99 (0.69—1.43)	0.97
≥ 13 academic	0.98 (0.71—1.33)	0.88	0.72 (0.52—1.00)	0.051
Population group				
Jews	1		1	
Arabs	1.33 (0.94—1.89)	0.11	0.84 (0.58—1.22)	0.37
Socioeconomic status				
Above poverty line	1.14 (0.87—1.50)	0.34	1.05 (0.79—1.39)	0.74
Below poverty line	1		1	
Subjective Health status				
Good	1		1	
Poor	2.47 (1.99—3.06)	< .0001	2.56 (2.03—3.23)	< .0001
Comorbidity				
No chronic illness	0.25 (0.18—0.35)	< .0001	0.28 (0.16—0.48)	< .0001
1–3 chronic illnesses	1		1	
≥ 4 chronic illnesses	6.36 (3.85—10.50)	< .0001	3.23 (2.40—4.33)	< .0001

Models were adjusted to survey, sex, age group, education, population group, socioeconomic status, subjective health status, and comorbidity

### Medications taken and their indications for use

The drugs taken according to the ATC classification are described in Table 4, as percent of total drugs taken in each survey. The majority of the drugs belong to ATC groups A, B, C, M and N: **A**limentary tract and metabolism, **B**lood and blood forming organs, **C**ardiovascular system, **M**usculoskeletal system and **N**ervous system, respectively. These classes represent 67.3% and 68% of all drugs taken in MZ1 and MZ2, respectively. The other drugs taken (not shown) were those taken for eyes, urinary tract disturbances, dermatological conditions, gastrointestinal disorders, respiratory disorders, inflammatory conditions, and hormonal needs. In both surveys**,** the most prevalent reported medications were cardiovascular medications such as ACE inhibitors and statins, being prescribed to over one-third of the participants (36.7%, 37.7%); anticoagulation drugs ranked second highest, being prescribed to about one-tenth of participants. Other commonly reported medications belonged to diabetes, and H2 Blocker categories. We noted an  increase in MZ2 for A02, A10, C09, C10 and a decrease in MZ2 for the rest of the medications (*p* < 0.05).

**Table 4:** Medications taken by Israeli older adult participants according to survey

ATC	Group name	Mabat Zahav 1 *N* = 10,012 drugs		Mabat Zahav 2 *N* = 4551 drugs		*P* –value
		**n**	**% of total**	**n**	**% of total**	
**A**	**Alimentary tract and metabolism:**					
A02	Drugs for acid related disorders	471	4.7	255	5.6	0.021*
A10	Drugs used in Diabetes	640	6.4	337	7.4	0.024*
**B**	**Blood and blood forming organs:**					
B01	Anti- Thrombotic Agents	922	9.2	444	9.8	0.29
**C**	**Cardiovascular system:**					
C02	Antihypertensives	162	1.6	57	1.3	0.10
C03	Diuretics	533	5.3	160	3.5	< .0001*
C07	Beta Blocking Agents	692	6.9	296	6.5	0.36
C08	Calcium Channel Blockers	552	5.5	232	5.1	0.30
C09	Agents acting on the Renin-Angiotensin System	788	7.9	407	8.9	0.025*
C10	Lipid modifying agents	976	9.8	562	12.4	< .0001*
**M**	**Musculo-skeletal system:**					
M01	Anti-inflammatory and Anti-rheumatic Products	241	2.4	41	0.9	< .0001*
M05B	Drugs Affecting Bone Structure and mineralization	213	2.1	40	0.9	< .0001*
**N**	**Nervous system:**					
N02AA	Natural opium alkaloids	122	1.2	53	1.2	0.78
N05CD	Sleeping	288	2.9	121	2.7	0.47
N06A	Antidepressants	151	1.5	97	2.1	0.007*

The certain drug classes represent 67.3% and 68% of total drugs taken in MZ1 and MZ2, respectively

*ATC* Anatomical Therapeutic Chemical classification of drugs

^*^P-value for the differences between surveys (Mabat Zahav 1 and 2), examined by Chi-square test. *Significant difference according to the Benjamini–Hochberg procedure (BH step-up procedure) controls the false discovery rate (FDR) at level alpha = 0.05

Of the drugs reported, for 10.2% in MZ1 and 8.3% in MZ2, the indication was not known by the interviewee. In addition, misclassifications were found, i.e., the stated reason was not correct, for 2.7% and 3.2% of drugs in MZ1 and MZ2, respectively.

## Discussion

This study describes the scope of the polypharmacy phenomenon and its associated factors in representative samples of Israeli community dwelling older adults, based on two national surveys conducted in the years 2005–6 and in 2014–15. Adjusted to possible risk factors, including basic demographic and health characteristics, multiple medication use was reduced significantly between the two surveys, but is still highly prevalent (56.3%). Polypharmacy was higher in women, and positively associated with increases in age, comorbidity of chronic illnesses, and reported poor health. No significant associations were found with sex or with socioeconomic background.

### Prevalence of polypharmacy

Comparing the prevalence rates of polypharmacy, the rates found in this study were in accordance to those reported in several studies, using similar samples and definitions of polypharmacy [11, 21, 22]. According to data from the Slone Survey [22], the prevalence of people aged 65 and over in the USA using five or more drugs (including OTC) was 58%. Junius-Walker et al. reported an average of 5.1 drugs (including OTC) and polypharmacy rates of 53.7%, in 466 ambulatory patients aged over 70 years who were randomly selected from two areas of Germany [21]. In some countries, higher rates were reported [23, 24] than those in Israel, whereas in others, they were lower [25, 26]. The lack of standardization in definitions of polypharmacy and methods of measurement and/or documenting makes it very difficult to compare data between surveys, and explain these differences.

The high prevalence of drug use in the Israeli older adult population reflects the increase in life expectancy and the rapid growth of this population in Israel. Medication treatment for the elderly in the community was discussed extensively since 2003 at the National Council for Geriatrics, and in 2007 the issue of "Standards for Clinics for Comprehensive Geriatric Assessment in the Community", was published by the Israeli Ministry of Health [27]. In the 2010 Israel State Comptroller’s Report, a special chapter was devoted to polypharmacy in the elderly [14]. These efforts may explain the decrease found in polypharmacy observed in this study in 2014–15. Still, the fact that 56% were with polypharmacy stresses the need for optimization of the prescriptions.

### Determinants of polypharmacy

Polypharmacy conditions were significantly associated with older age, health status, and comorbidity of chronic illnesses, findings that are consistently reported [11, 28–31]. This reflects the increased prevalence with aging of diseases that require therapy, and the difficulty in stopping usage of a medication, once it has been prescribed, resulting in cumulative prescriptions [25]. Women were found to be more likely to be exposed to polypharmacy, as previously reported [11, 19, 31]. It is thought to be related to the fact that females are more concerned about their health, more likely to consult doctors earlier than males (when symptoms begin), and are more accustomed to taking medications [32]. However, no association was found between sex and major polypharmacy. Further research exploring the relationship between sex and polypharmacy is warranted [32]. Reduced education level versus academic level was found to be associated with major polypharmacy, as those in a lower socioeconomic level, generally, seemed to be more susceptible to polypharmacy [30]. However, in this study no association was found. This is possibly explained by the Israeli healthcare system, which provides universal population coverage. The "State Health Insurance" Law mandates a list (basket) of medical services and medicines, which must be provided, and this list is updated annually.

### Classes of medications

Cardiovascular system drugs were the most frequent therapeutic class, a finding in line with previous studies [25, 28, 33]. The most common medications reported were for hypertension, followed by those for lowering cholesterol. This is similar to the pattern reported in the Slone Survey, where the five most common reasons were hypertension (13%), pain (7.7%), cholesterol (7.4%), heart (6.9%) and headache/migraine (5.6%) [22]. The reason that “pain” was so prevalent is that they did not exclude drugs taken for short-term pain relief, as was done in our study. According to the NHANES 2015–16 survey [10], in adults aged 60–79, the most commonly used prescription drugs were lipid-lowering drugs (45.0%), antidiabetic agents (23.6%), beta-blockers (for high blood pressure or heart disease, 22.3%), ACE inhibitors (21.3%), and proton pump inhibitors (16.9%).. This distribution is similar to other reports [34, 35]. In our surveys, the prevalence of antacid use was about 5%, and this may represent differences in prescribing patterns. Some differences may be due to different policies regarding optimal drug regimens for treating certain conditions, and different pricing policies.

Compared to the prevalence of drugs used in the 2005–2006 survey, in 2014–2015 a significant increase in the prevalence of anti-hyperlipidemic agents, in particular statins, was observed. The use of anti-diabetic, ACE inhibitors, proton-pump inhibitors, and antidepressants agents was also increased, and these trends were reported previously, i.e., in the Netherlands and the United States [36]. The use of osteoporosis treatments was decreased. This change is consistent with the significant decrease in the prevalence of osteoporosis reported in MZ2 versus MZ1 (16.5% and 27.0%, respectively, *p* < 0.0001).

### Strengths and limitations

Among the major strengths of this study is primarily the large-scale, nationally representative samples of the community-dwelling older adults in Israel. Secondly, the most recent data available were used, including a variety of demographic and health characteristics and construct validity questions and tests. These enabled controlling for various factors that could potentially confound associations with polypharmacy. Thirdly, the similar tools used in both surveys enabled examination of the changes in medication use over a decade.

The study also has some limitations. First, a causal relation between the factors examined and medication use cannot be assumed, due to the cross-sectional design of both surveys. Secondly, the study is based on self-reported data, which may be subject to social desirability response bias. However, self-reported medication use has been shown to be one of the most reliable ways of ascertaining medication uses (including OTC drugs) taken by the elderly [35, 37]. A very small percentage reported taking medications on a regular basis, but nevertheless refused to bring their drugs. However, this was more than offset by the study methodology, which insisted that the interviewees produce their actual medications rather than just remember them.

## Conclusions and recommendations

The use of multiple medications was seen in both surveys with similar drug taking patterns. This is of concern, particularly in view of the lack of knowledge as to indications in some 10% of the population, which, in turn, can lead to adverse side effects and incorrect usage. Concerted efforts should be made in the health care system to ensure 1) that all patients, especially the elderly, understand and adhere to their appropriate medication regimens and 2) treating physicians and health teams assess regularly and reduce, where possible, the number of medications.

In order to monitor polypharmacy, and possibly reduce its incidence and possible side effects, it is recommended that a routine re-examination of medications in selected patients may improve adherence [37] and by extension will enhance their health. The recent WHO report suggests the following plan of action for monitoring polypharmacy [3]: all patients with 10 or more regular medicines, taken every day or every week. Alternatively, reviewing patients receiving between four and nine regular medicines who also: have at least one prescribing issue that meets the criteria for potentially inappropriate prescribing; have evidence of being at risk of a well-recognized potential drug–drug interaction or a clinical contraindication; have evidence from clinical records of difficulties with taking medicines, including problems with adherence; have no or only one major diagnosis recorded in the clinical record since many medications are unlikely to be justified in patients without multiple clinical conditions; or are receiving end-of-life or palliative care. To this list, we may add older adult patients with frailty and those who have had at least one hospitalization in the previous year.

The study did not attempt to address the *appropriateness* of the medications being taken. This important issue should be tackled in the future to analyze medications taken as compared to self-reported medical conditions. Appropriate medication prescribing and adherence is the key to therapeutic success [38].

In addition to establishing good practitioner-patient communication, another effective intervention is better drug labeling from the drug pharmacies, e.g. color-coded labeling and large succinct written instructions – on the drug itself and/or the pillboxes [38]. From the patient's point of view, a helpful accessory might be a "Pill Card" linked to a pill organizer, containing information about the medication names and indications, in addition to when and what dosage to take. This would be generated by the physician, checked by the dispensing pharmacist, and re-checked/altered as necessary, at the next physician visit. During this process medications can be optimized with the constant aim to promote patient safety by deprescribing and re-prescribing as appropriate and as necessary [39].

## Data Availability

Raw data of the first 'Mabat Zahav' survey are available at: https://www.health.gov.il/UnitsOffice/ICDC/mabat/Pages/Mabat_Gold.aspx. The datasets generated during and analyzed during the second 'Mabat Zahav' survey are not publicly available as they are currently identifiable but are available, in a non-identifiable data file from the corresponding author on reasonable request, pending approval of the Publications Committee of the Israel Center for Disease Control. The Israel Center for Disease Control is the research unit of the Israeli Ministry of Health, which is responsible for collecting and publishing data on the health of the population.

## Abbreviations

OTC: Over the counter
MZ1: Mabat Zahav 1
MZ2: Mabat Zahav 2
ATC: Anatomical Therapeutic Chemical
CBS: Central Bureau of Statistics
SES: Socioeconomic status
